# Prediction of random-regression coefficient for daily milk yield after 305 days in milk by using the regression-coefficient estimates from the first 305 days

**DOI:** 10.5713/ajas.17.0861

**Published:** 2018-03-13

**Authors:** Takeshi Yamazaki, Hisato Takeda, Koichi Hagiya, Satoshi Yamaguchi, Osamu Sasaki

**Affiliations:** 1 Dairy Cattle Group, Division of Dairy Production Research, Hokkaido Agricultural Research Centre, NARO, Sapporo 062-8555, Japan; 2 Animal Breeding Unit, Division of Animal Breeding and Reproduction Research, Institute of Livestock and Grassland Science, NARO, Tsukuba 305-0901, Japan; 3 Department of Life and Food Science, Obihiro University of Agriculture and Veterinary Medicine, Obihiro 080-8555, Japan; 4 Computing Section, Milk Recording Division, Hokkaido Dairy Milk Recording and Testing Association, Sapporo 060-0004, Japan

**Keywords:** Dairy Cattle, Lactation Curve, Milk Yield, Random Regression Model

## Abstract

**Objective:**

Because lactation periods in dairy cows lengthen with increasing total milk production, it is important to predict individual productivities after 305 days in milk (DIM) to determine the optimal lactation period. We therefore examined whether the random regression (RR) coefficient from 306 to 450 DIM (M2) can be predicted from those during the first 305 DIM (M1) by using a RR model.

**Methods:**

We analyzed test-day milk records from 85,690 Holstein cows in their first lactations and 131,727 cows in their later (second to fifth) lactations. Data in M1 and M2 were analyzed separately by using different single-trait RR animal models. We then performed a multiple regression analysis of the RR coefficients of M2 on those of M1 during the first and later lactations.

**Results:**

The first-order Legendre polynomials were practical covariates of RR for the milk yields of M2. All RR coefficients for the additive genetic (AG) effect and the intercept for the permanent environmental (PE) effect of M2 had moderate to strong correlations with the intercept for the AG effect of M1. The coefficients of determination for multiple regression of the combined intercepts for the AG and PE effects of M2 on the coefficients for the AG effect of M1 were moderate to high. The daily milk yields of M2 predicted by using the RR coefficients for the AG effect of M1 were highly correlated with those obtained by using the coefficients of M2.

**Conclusion:**

Milk production after 305 DIM can be predicted by using the RR coefficient estimates of the AG effect during the first 305 DIM.

## INTRODUCTION

Daily milk yields of Holstein cow generally peak at 1 or 2 months after calving and gradually decline thereafter. Dairy cows must conceive during lactation and reach next calving to maximize their high-yield period. In general, the economically optimal lactation period and calving interval are considered to be around 10 and 12 months, respectively. However, because milk production per cow increases over decades [1], a lactation period longer than 10 months is more appropriate for cows with high peak yield and prolonged lactation persistency after the lactation peak [2,3]. Currently lactations in an estimated more than 30% of dairy cows extend beyond 305 days (i.e., 10 months) [e.g., 4,5], because lactation periods in cows lengthen with increasing milk production and decreasing reproductive performance [6,7]. The average lactation period for Japanese Holsteins was 366 days in 2016 [1]. The optimal lactation period for Japan’s current dairy cow population should be determined.

The optimal lactation period for individual cows depends on their total milk yields during lactation or during their lifetimes, including the milk yields after 305 days after calving. In most countries, individual production abilities have been expressed as 305-day cumulative yield, which is predicted by using the lactation curve within the 305 days in milk (DIM), in accordance with the Interbull (International Bull Evaluation Service) and International Committee for Animal Recording (ICAR) guidelines [8,9]. If the lactation curve and milk yields after 305 DIM can be predicted by using the lactation curve during 305 DIM, the lactation productivities with various shapes of lactation curves, e.g. peak yield or lactation persistency, can be predicted by using individual lactation curve estimates. Predicting the milk yields after 305 DIM or the lactation yields of individual cows is important for determining the timing of insemination or drying off. To our knowledge, the feasibility of predicting milk yield after 305 DIM by using the parameters obtained during the first 305 DIM has not yet been assessed. Haile-Mariam and Goddard [10] reported that the genetic correlations among the test-day (TD) milk yields of the first, second, and third 200 DIM were high (0.83 to 0.93) in both the first and second lactations. These results suggest that milk yields after 305 DIM might be predicted by using those obtained before 305 DIM.

A random regression (RR) TD model is more accurate than a lactation model in accounting for the additive genetic (AG) and permanent environmental (PE) effects on lactation curve shape [e.g., 11,12]. In many countries, RR-TD models incorporating the RR coefficients for AG effect are used to estimate the breeding value of 305-day cumulative yield [13]. By extension, the RR coefficients for AG and PE effects during the first 305 DIM might be useful for estimating those after 305 DIM. Consequently, using the RR coefficients estimated for after 305 DIM to predict daily milk yields after 305 DIM might be more accurate than using the estimates of effects during 305 DIM.

Therefore, our objectives here were i) to investigate the relationships between the RR coefficients for TD milk yields during and after 305 DIM in the first and later lactations of Holstein cows and ii) to examine whether these RR coefficients and daily milk yield after 305 DIM could be predicted from those obtained during the first 305 DIM.

## MATERIALS AND METHODS

### Data

**Figure 1:**
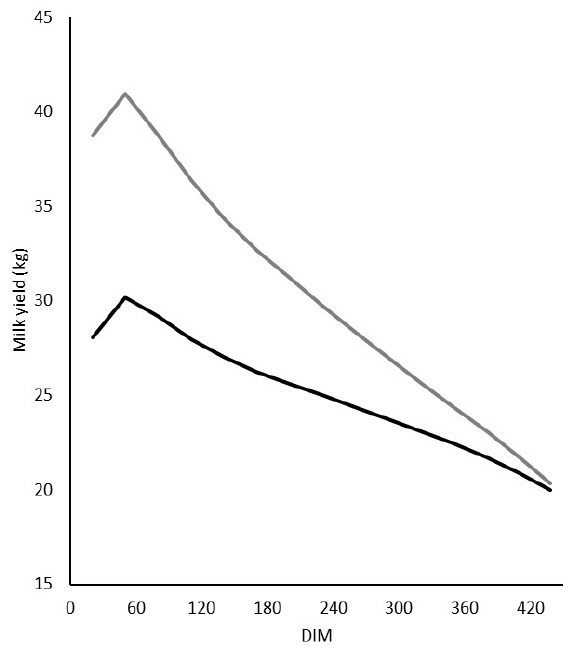
Average daily milk yields every 30 successive days in milk (DIM) for the first (black line) and later (gray line) lactations.

Monthly TD milk records through 450 DIM from 85,690 cows in their first lactations and 131,727 cows in their later (second to fifth) lactations in the Hokkaido region that had calved from 1998 through 2010 were obtained from the Hokkaido Dairy Milk Recording and Testing Association (http://www.hmrt.or.jp/sosik.html, accessed Oct. 26, 2017). Only the milk records of cows with a lactation length of more than 451 days, at least eight TD records during the first 305 DIM, at least three records from 306 to 450 DIM, and at least one record after 451 DIM were used for analysis. Pedigree records were traced back at least five generations. The average lactation curves of the first and later lactations are shown in Figure 1.

### Models

The data within the first 305 DIM (M1) and from 306 to 450 DIM (M2) during the first and later lactations were analyzed separately by using different single-trait RR animal models.

Model_M1, which was applied to TD milk records in M1 (Y1), was

$$\text{Y}1_{ijkl}={\text{TYM}}_{i}+\sum_{m=0}^{6}{\text{b}}_{jm}\text{w}{\left(t_{kl}\right)}_{m}+\sum_{m=0}^{2}{\text{u}}_{km}\text{w}{\left(t_{kl}\right)}_{m}+\sum_{m=0}^{2}{\text{p}}_{km}\text{w}{\left(t_{kl}\right)}_{m}+{\text{e}}_{ijkl}$$

**Figure 2:**
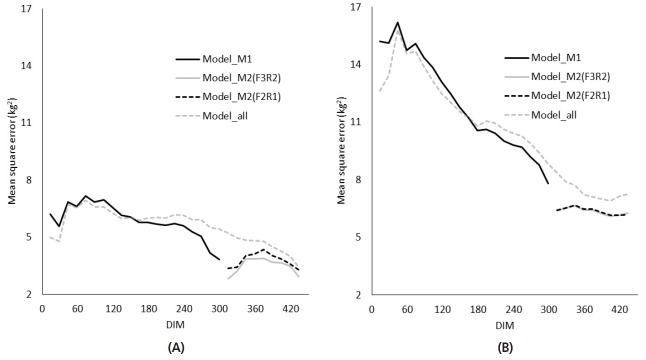
Mean square error every 15 successive days in milk (DIM) for the first (A) and later (B) lactations in different models: Model_M1 (black solid line), which was applied to the milk records during the first 305 DIM (M1); Model_M2, which was applied to the milk records from 306 to 450 DIM (M2) with the covariates of fixed and random regression as third- and second-order Legendre polynomials (F3R2, gray solid line) and as second- and first-order Legendre polynomials (F2R1, black dashed line), respectively; and Model_all (gray dashed line), which was applied to the total milk records during the first 450 DIM.

Where TYM*_i_* is the fixed test-year–month effect *i*; b*_jm_* is the *m*th fixed regression coefficient specific to parity *j* (one level for the first lactation, and four levels each for the second through fifth lactations); u*_km_* and p*_km_* are mth RR coefficients specific to cow *k* for AG and PE effects, respectively; w(t*_kl_*)*_m_* is a covariate associated with DIM *t_kl_* for TD record *l* of cow *k*; and e*_ijkl_* is a random residual effect associated with Y1. The covariates of the fixed regression coefficient for parity effect are fifth-order Legendre polynomials [14], with the exponential term of the Wilmink function (e^−0.05t^) as a sixth-order covariate [15,16]. The covariates of the RR coefficients for AG and PE effects are second-order Legendre polynomials [17,18] in accordance with the official genetic evaluation model for production traits in Japan [19]. Generally, herd effect for TD records was included in the RR-TD model. Because only the records of cows with extremely long lactations (i.e., more than 451 days) were used for analysis, it was difficult to make a contemporary group of each herd and to reliably estimate herd effects and AG effects simultaneously in our preliminary study. Therefore, we did not account for herd effect in the model. The mean square errors that we obtained (Figure 2) were similar to the residual variances reported by Bohmanova et al [5] which account for herd effect. Therefore, we consider that the estimation accuracies of the models in our current study are similar to those in another study [5] that accounted for herd effect.

Model_M2, which was applied to TD milk records in M2 (Y2), was

$$\text{Y}2_{ijkl}={\text{TYM}}_{i}+\sum_{m=0}^{p}{\text{b}}_{jm}\text{w}{\left(t_{kl}\right)}_{m}+\sum_{m=0}^{q}{\text{u}}_{km}\text{w}{\left(t_{kl}\right)}_{m}+\sum_{m=0}^{q}{\text{p}}_{km}\text{w}{\left(t_{kl}\right)}_{m}+{\text{e}}_{ijkl}$$

Where the definitions of elements are the same as those described earlier for Model_M1. We set two combinations of the orders for the covariates of fixed (*p*) and random (*q*) regressions in Model_M2; the covariates of fixed and RR are second- and first-order Legendre polynomials (F2R1) and third- and second-order Legendre polynomials (F3R2), respectively.

Model_all, which applied to the whole TD milk records within the first 450 DIM (YA), was

$${\text{YA}}_{ijkl}={\text{TYM}}_{i}+\sum_{m=0}^{7}{\text{b}}_{jm}\text{w}{\left(t_{kl}\right)}_{m}+\sum_{m=0}^{3}{\text{u}}_{km}\text{w}{\left(t_{kl}\right)}_{m}+\sum_{m=0}^{3}{\text{p}}_{km}\text{w}{\left(t_{kl}\right)}_{m}+{\text{e}}_{ijkl}$$

Where the definitions of elements are the same as those for Model_M1 and Model_M2, as discussed. The covariates of the fixed regression are sixth-order Legendre polynomials, with the exponential term of the Wilmink function as the seventh-order covariate, and the covariates of the RR are third-order Legendre polynomials.

The covariance structures for all models were defined as

$$\text{var}\left[\begin{array}{c} u\\ p\\ e \end{array}\right]=\left[\begin{array}{ccc} G⊗A & 0 & 0\\ 0 & P⊗I & 0\\ 0 & 0 & R \end{array}\right]$$

Where **G** and **P** are AG and PE (co)variance square matrices, respectively, of RR coefficients; ⊗ is the Kronecker product; **A** is the AG relationship for animals; **R** is the identity matrix for cows; and **R** is a diagonal matrix of residual variance for each record. The DMU program [20] was used for REML to estimate the variance components and obtain the solutions of the regression coefficients for AG and PE effect. Mean square errors ($\sum_{i=1}^{n}{\left({\text{Y}}_{i}-{\hat{\text{Y}}}_{i}\right)}^{2}/n$) of every 15 successive DIM for Model_M1, Model_M2, and Model_all were compared.

### Predicting RR coefficients and daily milk yields in M2 from those in M1

Correlation coefficients among the RR coefficients for the AG and PE effects of Model_M1 and Model_M2 during the first and later lactations were calculated. We then performed a multiple-regression analysis of the combined RR coefficients of M2 on the RR coefficients of M1 during the first lactation and later lactations. Combined RR coefficients were defined as the values in which each dimensional regression coefficient of AG effect was added to that of the PE effect (i.e., u*_m_*+p*_m_* of Model_M2). We set two regression equations: the equation of combined RR coefficients of M2 on the RR coefficients for AG effect of M1 (REG_1) and that on the coefficients for AG and PE effects of M1 (REG_2). Multiple regression analysis was performed by using the REG procedure of the SAS software package [21]. The predictive values for daily milk yields of several DIM in M2 were calculated by using the combined RR coefficients predicted from REG_1 and REG_2, and the correlation coefficients between these values and those obtained by using the combined RR coefficients of Model_M2 were calculated. In addition, the predicted daily milk yields in M2 were calculated by using these combined RR coefficients and the solutions of fixed test-year–month effects and fixed regression coefficients. The correlation coefficients between these values and TD milk yields were calculated for every 15 successive days of M2.

## RESULTS AND DISCUSSION

### Comparison of errors among different models

The mean square errors every 15 DIM in M1 for Model_all were more than 5% larger than those for Model_M1 at the same DIM after 186 days in the first lactation (Figure 2A) and after 201 days in later lactations (Figure 2B) even though the order of RR coefficients of Model_all was highest among the models in this study. Those in M2 for Model_M2 were smaller than those for Model_all at the same DIM in all lactations. The mean square errors that we obtained during the first 305 DIM in the first lactation were similar to the residual variances reported by Bohmanova et al [5]. In that study, the authors applied an RR model and found that the residual variances from 275 to 305 DIM that were estimated by using TD records from the first 365 DIM in the first lactation were larger than those obtained by using the records from the first 305 DIM.

The mean square errors every 15 DIM in M2 for Model_M2 (F3R2) were smaller than those for Model_M2 (F2R1) in the first lactation (Figure 2A). However, in later lactations (Figure 2B), in which the lactation curves were more variable than that for the first lactation, these M2 errors did not differ between Model_M2 (F3R2) and Model_M2 (F2R1). Therefore, we decided to use Model_M2 (F2R1) for the RR model in M2.

### Relationships between RR coefficients in M1 and M2

Table 1 contains the descriptive statistics values for the RR coefficients for the AG and PE effects of M1 estimated by using Model_M1 and those of M2 estimated by using Model_M2 (F2R1) during the first and later lactations. In both M1 and M2, the intercept for the AG effect had the largest standard deviation. The averages of the coefficients for PE effect were nearly 0.

The intercepts and second-order coefficients for the AG effects of M1 showed moderate to strong correlations with all of the RR coefficients for AG effect and with the intercepts for the PE effect of M2 in both the first and later lactations (Table 2). In addition, the first-order coefficients for the AG effect of M1 were moderately well correlated with these same coefficients of M2 during the first lactation, but the correlations were quite weak in later lactations. The correlations between all RR coefficients for the AG effect of M1 and the first-order coefficients for the PE effect of M2 and those between all RR coefficients for the PE effect of M1 and those for the AG and PE effects of M2 were very weak in all lactations. Haile-Mariam and Goddard [10] reported that the genetic correlation between the intercept for the RR coefficient of milk yield in the first 300 DIM and that in the second 300 DIM (from 301 to 600 DIM) in the first parity was 0.85. Our current results are in line with these previous findings.

The moderate to strong correlations between the intercept for the AG effect of M1 and all of the coefficients for the AG effect or the intercept for PE effect of M2 indicate that the value of the intercept for AG effect in M1 (i.e., the mean of the lactation curve for the individual AG effect during the first 305 DIM) affects the shape of the lactation curve for the AG effect and the intercept of the lactation curve for the individual environmental effect in M2. In addition, the second-order coefficients for the AG effect of M1 were significantly correlated with these coefficients of M2. However, because the standard deviations for the second-order coefficient for the AG effect (Table 1) were much smaller than those for the intercept, the influence of these coefficients on the RR coefficients of M2 could be much less than that of the intercept of M1. The lack of a relationship between the RR coefficients for the AG effect of M1 and the first-order coefficient for the PE effect of M2 indicates that the shape of the lactation curve for an individual AG effect in M1 does not affect the slope of that for an individual environmental effect in M2. Furthermore, the RR coefficients for the PE effect of M1 showed no correlation with those for either the AG effects or the PE effects of M2. This result suggests that the differences between the shapes of the lactation curves for individual environmental effects of M1 do not affect those of M2.

### Predicting RR coefficients and daily milk yields in M2 from those in M1

We performed a multiple regression analysis by using the combined RR coefficients of the AG and PE effects of M2 as objective variables, because the variances for each dimensional RR coefficient were too small to predict individually, except in the case of the intercept of AG (Table 1). The coefficients of determination (*R*^2^) for REG_1 and REG_2 of the combined intercepts of M2 were much higher than those for the combined first-order coefficients of M2 (Table 3). The differences of *R*^2^ between REG_1 and REG_2 for the same objective variables were small; for the intercept and first-order coefficient, these values were 0.022 and 0.011, respectively, in the first lactation and 0.046 and 0.024 in later lactations. The small differences in *R*^2^ arose from the very weak correlations between all of the RR coefficients for the PE effect of M1 and those for the AG and PE effects of M2 (Table 2). Therefore, we considered that the RR coefficients for the PE effect of M1 had little effect as explanatory variables in the multiple-regression equation for predicting the RR coefficients of M2. The standardized partial regression coefficients for the intercept for the AG effect of M1 were much larger than those for the other explanatory variables in the equation of the combined intercepts of M2 (Table 4). This result indicates that the intercepts for the AG effect of M1 have a large effect in the equations that predict the RR coefficients of M2.

Strong correlations emerged at 335, 365, 395, and 425 DIM between the predictive values for daily milk yield calculated by using the combined RR coefficients of Model_M2 and those estimated by using the combined RR coefficients predicted from REG_1 or REG_2 (Table 5). In particular, the correlation coefficients at 335 DIM were highest (from 0.824 to 0.889); the values then gradually declined with increasing DIM (e.g., those at 425 DIM ranged from 0.778 to 0.839). The differences between the correlations for REG_1 and REG_2 at the same DIM were small, ranging from 0.012 to 0.016 in the first lactation and from 0.018 to 0.026 in later lactations. We considered that this high prediction accuracy was due to the high prediction accuracies for the combined intercepts of M2 (Table 3), which had large variances (Table 1), despite the low accuracies for the combined first-order coefficients of M2. Moreover, the slopes of the lactation curves after about 200 DIM were relatively small (Figure 1), so the predictive errors for the first-order coefficients of M2 had less of an effect on the accuracies of the predictive values for daily milk yield than those for the intercepts of M2.

In addition, strong correlations (calculated for every 15 successive days of M2) emerged between the true TD milk yields and the predicted dairy milk yields obtained by using combined RR coefficients of Model_M2, REG_1, and REG_2 (Table 6); furthermore, the differences between the correlations for REG_1 and REG_2 at the same DIM were small. The correlations with the predicted milk yields of Model_M2 were fairly strong and constant, ranging from 0.928 to 0.948 in the first lactation and from 0.906 to 0.916 in the later lactations. The correlation coefficients with the predicted milk values of REG_1 or REG_2 from 306 to 320 DIM were highest (from 0.853 to 0.792), and the values gradually declined with increasing DIM. However, strong correlations (greater than 0.7) were maintained until 440 DIM in the first and later lactations and until 395 DIM in later lactations. Thus our current results suggest that the differences among individual daily milk yields after 305 DIM can be predicted by using the RR coefficient for the AG effect within 305 DIM.

In conclusion, first-order Legendre polynomials were the practical covariates of RRs for milk yields from 305 to 450 DIM in the random-regression TD model. Moderate to strong correlations emerged between the RR coefficients for AG effect or between the intercept for the PE effect from 305 to 450 DIM and the intercepts for AG effect within the first 305 DIM; the mean and slopes of the individual lactation curves after 305 DIM depended on the mean of the lactation curve for the AG effect within the first 305 DIM. The *R*^2^ for multiple regression of the RR coefficients after 305 DIM on those within the first 305 DIM were moderate to high when the intercepts after 305 DIM were the objective variables. The predictive values for daily milk yield after 305 DIM that were obtained by using the random-regression coefficients for AG effect within the first 305 DIM were highly correlated with those calculated by using the coefficients after 305 DIM. These results suggest that milk production after 305 DIM can be predicted by using the random-regression coefficient estimates for AG effect within the first 305 DIM. Combining these random-regression coefficients with the fixed regression coefficients of the lactation curve related to an individual cow’s environment (e.g., herd or calving season) will provide an estimate of the total milk yield during that cow’s lactation. Predicting the milk yield after 305 DIM or the total lactation yield for individual cows will facilitate the timing of insemination or drying off to optimize individual lactation periods.

## Figures and Tables

**Table 1:** Averages and standard deviations (SDs) of solutions for regression coefficients for the additive genetic (AG) and permanent environmental (PE) effects during the first 305 DIM (M1) and from 306 to 450 DIM (M2) in the first and later lactations

Items			First lactation		Later lactations	
	
			Average	SD	Average	SD
M1	AG	Intercept	4.479	4.791	4.617	5.793
		First-order	0.370	0.762	−0.154	0.978
		Second-order	−0.683	0.876	−0.648	1.240
	PE	Intercept	0.000	0.640	0.000	0.577
		First-order	0.000	1.879	0.000	1.986
		Second-order	0.000	1.175	0.000	1.335
M2	AG	Intercept	4.092	4.259	3.515	4.194
		First-order	−0.336	0.319	−0.419	0.450
	PE	Intercept	0.000	0.885	0.000	1.025
		First-order	0.000	1.059	0.000	0.817

**Table 2:** Correlation coefficients between the solutions for the regression coefficients for the additive genetic (AG) and permanent environmental (PE) effects during the first 305 DIM (M1) and those from 306 to 450 DIM (M2) for the first and later lactations

Items			M1					

			AG			PE		
	
			Intercept	First-order	Second-order	Intercept	First-order	Second-order
First lactation								
M2	AG	Intercept	0.850	0.732	−0.779	0.066	0.126	0.042
		First-order	−0.752	−0.569	0.762	−0.065	−0.113	−0.039
	PE	Intercept	0.612	0.465	−0.602	0.062	0.125	0.030
		First-order	−0.050	−0.027	0.048	−0.018	0.004	−0.026
Later lactations								
M2	AG	Intercept	0.771	0.073	−0.730	−0.027	0.165	0.061
		First-order	−0.712	0.142	0.698	0.018	−0.139	−0.056
	PE	Intercept	0.527	−0.043	−0.510	−0.038	0.168	0.048
		First-order	−0.065	0.052	0.064	−0.032	0.033	−0.019

**Table 3:** Multiple regression equations predicting the combined random regression (RR) coefficients1) from 306 to 450 DIM (M2) obtained by using the RR coefficients for the additive genetic (AG) and permanent environmental (PE) effects during the first 305 DIM (M1) for the first and later lactations

Items			Equation predicting the combined RR coefficient of M2							

			First lactation				Later lactations			
	
			Intercept		First-order		Intercept		First-order	
			
			REG_12)	REG_23)	REG_1	REG_2	REG_1	REG_2	REG_1	REG_2
Coefficient of determination (*R*^2^)			0.743	0.765	0.068	0.079	0.653	0.699	0.146	0.170
Intercept			0.307	0.101	−0.075	0.003	−0.098	0.161	−0.142	−0.071
Partial regression coefficient on RR coefficients of M1	AG	Intercept	0.948	0.955	−0.296	−0.033	1.059	0.858	−0.051	−0.044
		First–order	2.005	1.176	0.031	0.155	1.628	0.890	0.001	0.123
		Second–order	1.762	1.059	0.205	0.365	1.582	0.726	0.068	0.198
	PE	Intercept	-	−0.795	-	0.498	-	−2.093	-	5.145
		First–order	-	0.230	-	0.091	-	0.616	-	0.710
		Second–order	-	0.616	-	−0.119	-	1.673	-	−1.265

1) Combined RR coefficients are defined as values obtained by adding each dimensional coefficient of the AG effect to that of the PE effect.

2) REG_1 is the multiple regression equation of the combined RR coefficients of M2 on the AG coefficients of M1.

3) REG_2 is the multiple regression equation of the combined RR coefficients of M2 on the AG and PE coefficients of M1.

**Table 4:** Standardized partial regression coefficients of multiple regression equations predicting the combined random regression (RR) coefficients1) from 306 to 450 DIM (M2), obtained by using the RR coefficients for the additive genetic (AG) and permanent environmental (PE) effects during the first 305 DIM (M1) in the first and later lactations

RR coefficient of M1		Equation predicting the combined RR coefficient of M2							

		First lactation				Later lactations			
	
		Intercept		First-order		Intercept		First-order	
			
		REG_12)	REG_23)	REG_1	REG_2	REG_1	REG_2	REG_1	REG_2
AG	Intercept	0.914	0.921	−0.124	−0.137	1.219	0.988	−0.299	−0.258
	First-order	0.308	0.180	0.021	0.103	0.316	0.173	0.001	0.123
	Second-order	0.311	0.187	0.157	0.279	0.390	0.179	0.086	0.251
PE	Intercept	-	−0.102	-	0.279	-	−0.240	-	3.025
	First-order	-	0.087	-	0.150	-	0.243	-	1.437
	Second-order	-	0.146	-	−0.123	-	0.444	-	−1.722

1) Combined RR coefficients are as defined in the footnote to Table 3.

2),3) REG_1 and REG_2 are as defined in the footnote to Table 3.

**Table 5:** Correlation coefficients at 335, 365, 395, and 425 days in milk (DIM) between the predictive values of daily milk yield calculated by using the combined random regression (RR) coefficients1) of Model_M22) and those obtained by using the combined RR coefficients predicted from multiple regression equations for the first and later lactations

DIM	First lactation		Later lactations	
	
	REG_13)	REG_24)	REG_1	REG_2
335	0.875	0.889	0.824	0.854
365	0.866	0.881	0.814	0.842
395	0.852	0.864	0.799	0.825
425	0.823	0.839	0.778	0.803

1) Combined RR coefficients are as defined in the footnote to Table 3.

2) Model_M2 is applied to the milk records from 306 to 450 DIM (M2), with the covariates of fixed and random regression as second and first-order Legendre polynomials, respectively.

3),4) REG_1 and REG_2 are as defined in the footnote to Table 3.

**Table 6:** Correlation coefficients of every 15 successive days from 306 to 450 days in milk (DIM) between true test-day milk yields and the predicted dairy milk yields by using combined random regression (RR) coefficients1) of Model_M22), REG_13), and REG_24) for the first and later lactations

DIM	First lactation			Later lactations		
	
	Model_M2	REG_1	REG_2	Model_M2	REG_1	REG_2
306–320	0.948	0.837	0.853	0.916	0.792	0.820
321–335	0.947	0.832	0.849	0.911	0.782	0.814
336–350	0.935	0.812	0.826	0.907	0.757	0.782
351–365	0.933	0.801	0.816	0.907	0.753	0.782
366–380	0.928	0.785	0.796	0.906	0.727	0.750
381–395	0.932	0.774	0.786	0.907	0.716	0.742
396–410	0.935	0.756	0.767	0.907	0.688	0.711
411–425	0.939	0.738	0.749	0.908	0.673	0.697
426–440	0.945	0.711	0.720	0.908	0.641	0.661
441–450	0.945	0.697	0.707	0.912	0.628	0.648

1) Combined RR coefficients are as defined in the footnote to Table 3.

2) Model_M2 is as defined in the footnote to Table 5.

3),4) REG_1 and REG_2 are as defined in the footnote to Table 3.

## References

[1] ICAR (2017). Milk recording surveys on cow, sheep and goats [Internet].

[2] Inchaisri C, Jorritsma R, Vos PLAM, van der Weijden GC, Hogeveen H (2011). Analysis of the economically optimal voluntary waiting period for first insemination. J Dairy Sci.

[3] Knight C (2005). Extended lactation: turning theory into reality. Adv Dairy Technol.

[4] VanRaden PM, Dematawewa CMB, Pearson RE, Tooker ME (2006). Productive life including all lactations and longer lactations with diminishing credits. J Dairy Sci.

[5] Bohmanova J, Miglior F, Jamrozik J (2009). Use of test-day records beyond three hundred five days for estimation of three hundred five-day breeding values for production traits and somatic cell score of Canadian Holsteins. J Dairy Sci.

[6] Hagiya K, Terawaki Y, Yamazaki T (2013). Relationships between conception rate in Holstein heifers and cows and milk yield at various stages of lactation. Animal.

[7] Haile-Mariam M, Pryce JE (2015). Variances and correlations of milk production, fertility, longevity, and type traits over time in Australian Holstein cattle. J Dairy Sci.

[8] Interbull (2001). Interbull guidelines for national and international genetic evaluation systems in dairy cattle with focus on production traits. Interbull Bulletin.

[9] ICAR (2016). ICAR rules, standards and guidelines for dairy production recording. In: International Agreement of Recording Practices, Section 2.

[10] Haile-Mariam M, Goddard ME (2008). Genetic and phenotypic parameters of lactations longer than 305 days (extended lactations). Animal.

[11] Jamrozik J, Schaeffer LR, Dekkers JCM (1997). Genetic evaluation of dairy cattle using test day yields and random regression model. J Dairy Sci.

[12] Jensen J (2001). Genetic evaluation of dairy cattle using test-day models. J Dairy Sci.

[13] Interbull (2017). National genetic evaluation forms provided by countries [Internet].

[14] Kistemaker GJ (2003). The Canadian test day model using Legendre polynomials. Interbull Bulletin.

[15] Wilmink JBM (1987). Comparison of different methods of predicting 305-day milk yield using means calculated from within-herd lactation curves. Livest Prod Sci.

[16] Schaeffer LR, Jamrozik J, Kistemaker GJ, Van Doormaal BJ (2000). Experience with a test-day model. J Dairy Sci.

[17] Gernand E, Waßmuth R, von Borstel UU, König S (2007). Heterogeneity of variance components for production traits in large-scale dairy farms. Livest Sci.

[18] Yazgan K, Makulska J, Weglarz A, Ptak E, Gierdziewicz M (2010). Genetic relationship between milk dry matter and other milk traits in extended lactations of Polish Holstein cows. Czech J Anim Sci.

[19] National Livestock Breeding Center (2018). Genetic evaluation for production traits [Internet].

[20] Madsen P, Jensen J (2013). A user’s guide to DMU [Internet].

[21] SAS Institute Inc (2015). SAS/STAT 141 User’s guide.

